# Systematic exploration of the mechanical properties of 13 621 inorganic compounds†
†Electronic supplementary information (ESI) available: Extra figures on the distribution of elastic moduli, list of materials with negative Poisson's ratio and negative linear compressibility. See DOI: 10.1039/c9sc01682a


**DOI:** 10.1039/c9sc01682a

**Published:** 2019-07-31

**Authors:** Siwar Chibani, François-Xavier Coudert

**Affiliations:** a Chimie ParisTech , PSL University , CNRS , Institut de Recherche de Chimie Paris , 75005 Paris , France . Email: siwar.chebbi@chimieparistech.psl.eu ; Email: fx.coudert@chimieparistech.psl.eu

## Abstract

In order to better understand the mechanical properties of crystalline materials, we performed a large-scale exploration of the elastic properties of 13 621 crystals from the Materials Project database, including both experimentally synthesized and hypothetical structures. We studied both their average (isotropic) behavior, as well as the anisotropy of the elastic properties: bulk modulus, shear modulus, Young's modulus, Poisson's ratio, and linear compressibility. We show that general mechanical trends, which hold for isotropic (noncrystalline) materials at the macroscopic scale, also apply “on average” for crystals. Further, we highlight the importance of elastic anisotropy and the role of mechanical stability as playing key roles in the experimental feasibility of hypothetical compounds. We also quantify the frequency of occurrence of rare anomalous mechanical properties: 3% of the crystals feature negative linear compressibility, and only 0.3% have complete auxeticity.

## Abstract 2

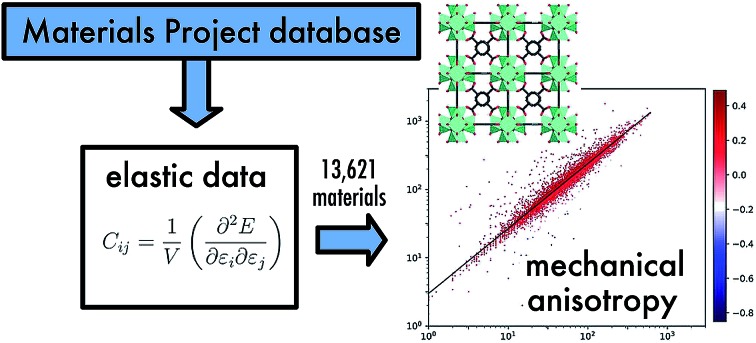
Exploring elastic properties of 13 621 crystals highlights how rare auxeticity and negative linear compressibility are.

## Introduction

The last decade has seen an incredible acceleration of the research into the links between structure and properties of crystalline materials, and in particular of several families of framework materials, including zeolites, metal–organic frameworks (MOFs), supramolecular frameworks, and dense inorganic materials. These have been studied by both experimental and theoretical methods, often in synergy, in order to provide a broad characterization of the properties of materials, and the links between their structure and properties—with the ultimate goal being to develop and design novel materials with targeted properties, or to identify as-yet unidentified properties of interest in known materials. Due to the important diversity and sheer number of known crystalline materials, several research groups have created different databases to systematically gather information from different sources.^1^–^6^ These databases are often classified according to the materials' class or chemical family: zeolites, zeolitic materials, metal–organic frameworks, *etc.*

Recently, a major development in the area of inorganic crystalline solids is the launch of the Materials Project (MP) database,^7^,^8^ a part of the Materials Genome Initiative whose goal is to apply high-throughput computing to map the properties of “all known inorganic materials”. This takes the form of an open database, which can be used both for data mining and interactive exploration, as well as open-source packages for developing analysing materials and their properties. The current release of the Materials Project database contains data derived from quantum mechanical calculations for more than 133 000 known and predicted inorganic compounds, and this number is rapidly growing. The data contained in the MP database include crystal structure, energy/formation enthalpy, electronic band structure, X-ray absorption spectra, synthesis descriptions extracted from (and linked to) the relevant publications, elastic tensors, piezoelectric constants, *etc.* A detailed description of the computational methods used to develop the database are available in ref. 9, for the interested reader. This initiative is proving popular, and more than 40 000 users have registered to the MP database, where they can interact online through the MP website (; https://www.materialsproject.org/), with no knowledge of programming required to run simple queries, or through a REpresentational State Transfer (REST) application programming interface (API).

One of the goals of the Materials Project is to accelerate the analysis and to allow users to access and interpret computational data. The existence of a well-documented public API allows this, and makes it possible to interface the MP database with other software. In this vein, our group has recently worked on the integration of an online application, named ELATE, allowing the analysis and the visualization of elastic tensors.^10^ The ELATE application interfaces two way with the Materials Project: you can search for compounds in the MP database from within ELATE, and you can use ELATE to analyse and visualise the elastic properties of compounds from the Materials Project website. We targeted elastic properties because they are key to the process of screening for physical and chemical properties, in order to ascertain the mechanical stability of materials during phases of discovery and design.^11^,^12^ Indeed, elastic constants are a linear response property, and as such can be characterized with comparatively cheap computational cost, and they provide a first-order approximation of the full response of materials to external mechanical constraints. In addition, they offer great potential for applications in mechanical energy storage,^13^ mechanochemistry,^14^ and geophysics.^15^

Despite the importance of elastic properties and mechanical stability of materials in screening for applications,^16^ it has only been characterized for a small fraction of all known inorganic compounds. Full experimental determination of the elastic constants, usually through single-crystal Brillouin scattering,^17^,^18^ is far from being a routine characterization, and thus predictive computational methods have been used as a complement. In 2015, Jong *et al.* calculated at the density functional theory (DFT) level the elastic properties for 1181 inorganic compounds,^19^ and uploaded the resulting data to the Materials Project database. This effort continued, using increasingly available high performance computing (HPC) resources, and at the time of writing the MP database contains elastic information for 13 621 inorganic compounds. In their seminal study, Jong *et al.* found a correlation between bulk modulus (*K*), shear modulus (*G*) and Poisson ratio (*ν*) for the 1181 compounds investigated, but the focus of their study concerned the calculation of elastic properties.^19^ Other correlations between mechanical properties have been uncovered: Pugh *et al.* found an interesting correlation between *K* and *G* moduli with hardness,^20^ while Snyder *et al.* related them with thermal conductivity.^21^

There is thus a real interest in a larger-scale investigation of the mechanical properties of inorganic compounds, in order to identify trends and find materials with anomalous mechanical properties (mechanical metamaterials^22^,^23^), such as negative Poisson's ratio (auxetic materials),^24^,^25^ or negative linear compressibility.^26^ These properties have been proposed for several applications: to make sensors and actuators, store mechanical energy, develop new materials with targeted mechanical responses.^27^ In this work, we report for the first time a mechanical properties investigation for 13 423 inorganic compounds, calculated at the quantum chemistry level and available in the Materials Project database. We provide first an analysis of the mechanical behavior of these materials in the isotropic approximation, looking at directionally-averaged properties such as bulk modulus and Young's modulus. We then perform a full tensorial analysis of their properties, looking at the shear modulus, Young's modulus, Poisson's ratio, and linear compressibility. We show that general mechanical trends, which hold for isotropic (noncrystalline) materials at the macroscopic scale, also apply “on average” for inorganic crystals. Further, we highlight the importance of elastic anisotropy and the role of mechanical stability as playing key roles in the experimental feasibility of hypothetical inorganic compounds. Finally, we quantify the frequency of occurrence of rare anomalous mechanical properties, such as negative linear compressibility and auxeticity.

## Computational methods

The tensor of elastic constants of a crystalline solid provides a good description of its response to external mechanical constraints, in the linear regime. When a stress *σ* is exerted on a material, it reacts by changing its shape and size, and this change is characterized by a strain *ε*. In the linear elastic regime, *i.e.* in the limit of small deformation, the stress and the strain can be linked through a generalized Hooke's law by a fourth-rank tensor, named the stiffness tensor *C* of second-order elastic constants: *σ* = *Cε*. In the Voigt notation, this stiffness tensor can be expressed as a 6 × 6 symmetric matrix of 21 independent elastic constants *C*_*ij*_.^28^ The crystal system of considered material yields additional symmetry constraints, further reducing the number of independent elastic constants: 3 for cubic crystals, 5 in the hexagonal case, 6 or 7 for the tetragonal classes, 9 for orthorhombic crystals and 13 for monoclinic crystals.^29^,^30^ These elastic constants can be calculated as second derivatives of the energy with respect to unit cell parameters:1
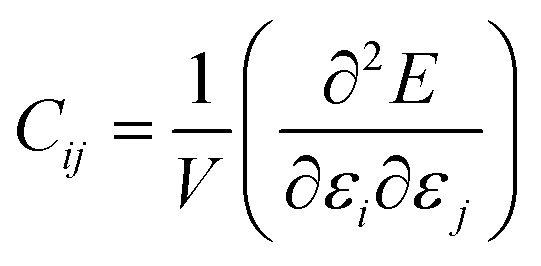
where the energy for each strained configuration can be evaluated through quantum chemistry calculations.

In the present study, we did not perform any explicit calculations of elastic constants, but used the second-order elastic tensors provided in the Materials Project database for 13 621 inorganic crystalline structures. This includes elastic tensors calculated by the group of de Jong *et al.*^19^ using the projector augmented wave (PAW) method implemented in the Vienna *Ab Initio* Simulation Package (VASP).^31^–^33^ All the data was obtained through the Materials Project API and the pymatgen package.^34^ Using these elastic tensors, we performed calculations of the physically meaningful quantities such as bulk modulus (*K*), shear modulus (*G*), linear compressibility (*β*), and Poisson's ratio (*ν*) through tensorial analysis, as described in Marmier *et al.*^35^ and implemented in the ELATE open source package.^10^ The source code used for the analysis, and the data gathered, are freely available online at ; https://github.com/siwar-chebbi.

## Results and discussion

### Elastic properties in the isotropic approximation

**Fig. 1:**
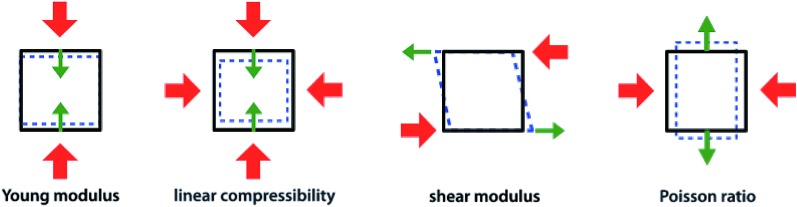
Representation (from left to right) of Young's modulus (*E*), linear compressibility (*β*), shear modulus (*G*), and Poisson's ratio (*ν*), four directional elastic properties of crystalline materials. Red arrows represent the direction of stress exerted, while green ones represent the axis along which the response is measured. Reproduced from ref. 10 with permission. Copyright 2016 IOP Publishing Ltd.

As stated above, the Materials Project database contains second-order elastic tensors, calculated through quantum-chemical methods, for 13 621 inorganic crystalline compounds to date. This tensor takes the form of a 6 × 6 symmetric matrix of second-order elastic constants, from which more physically meaningful properties can be calculated. The bulk modulus *K* is the simplest mechanical property of material; it is the inverse of the compressibility, and quantifies the resistance of the structure (measured by its volumetric strain) when it subjected to a isotropic stress (such as a hydrostatic pressure). The shear modulus *G*, named also the rigidity modulus, represents the resistance of a material under the influence of an opposing pair of shear stresses acting parallel to the material surface, see Fig. 1. *G* is a directional quantity, but for both *K* and *G* average values can be computer in the isotropic approximation, based on three different averaging schemes: Voigt,^36^ Reuss,^37^ and Hill.^38^ Voigt averaging assumes a uniform strain in a polycrystalline sample, while Reuss averaging assumes uniform stress; the Hill method corresponds to the arithmetic mean of the other two, and is considered the most accurate in a wide range of experimental conditions. Other average quantities available directly in the MP database are the elastic anisotropy and the average Poisson's ratio (*ν*) in the isotropic approximation—we refer the reader to the ref. 19 for details on their calculation.

To ensure the mechanical stability of a material, the values of *K* and *G* are required to be positive.^39^ From the original elastic properties information for 13 621 inorganic compounds contained in the MP database, we first applied stability criteria on *K* and *G* as a filter and found that 1312 structures (around 9% of materials in the database) were failing that test, *i.e.* computed to be mechanically unstable. This reflects the need for curation of computational data before it can be integrated into databases, or used for analysis. Moreover, we found some materials with reported values of *K* and *G* unphysically high. Given that diamond is one of the stiffest inorganic compounds, with *K* = 530 GPa and *G* = 440 GPa, we filtered out a handful of materials with *K* and *G* values larger than 10^3^ GPa. (A list of the 13 materials presenting *K* or *G* values larger than 10^3^ GPa is available in ESI,† with respective elastic properties values in the isotropic approximation.) We thus obtained a list of 12 296 crystalline compounds, of which 8335 have been experimentally synthesized and 3961 are hypothetical structures. We have then used the ELATE software package,^10^ available both as an open source Python module (available online at ; https://github.com/fxcoudert/elate) and as a web application, allowing 3D visualization with an open API (available at ; http://progs.coudert.name/elate). ELATE implements the tensorial analysis of second-order elastic constants, in the form of a 6 × 6 symmetric matrix in Voigt notation. It calculates: (i) average mechanical properties in the 3 averaging schemes, (ii) the eigenvalues of the elastic tensor (including softest and stiffest modes), (iii) minima and maxima of the elastic moduli with associated axes, (iv) 2D and 3D graphs of the spatial variations of all moduli. From 12 296 selected materials, based on Materials Project values of *K* and *G* within physical range, we found 216 materials with singular elastic matrix and 307 materials with at least one negative eigenvalue – indicating mechanical instability, a condition known as Born's criterion.^39^ For the rest of this paper, we restrict ourselves to the mechanically stable inorganic compounds, *i.e.*, 11 764 structures, of which 8050 have been experimentally synthesized and 3714 are hypothetical structures are considered.

**Fig. 2:**
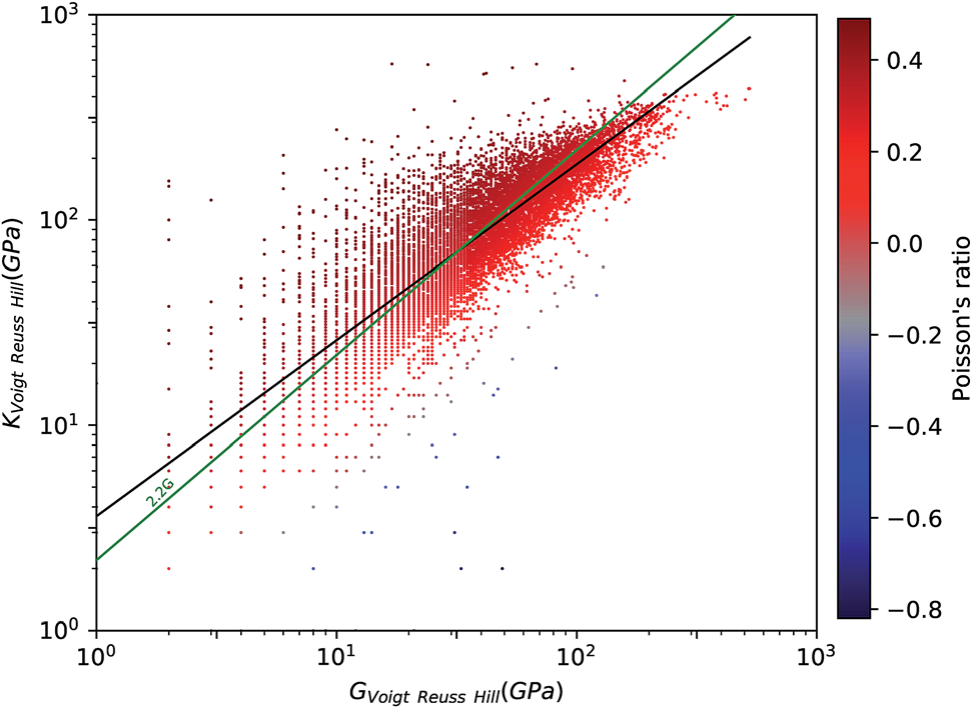
Plot of bulk modulus *K*_VRH_ against shear modulus *G*_VRH_ for 11 764 inorganic compounds from the Material Project database, using Voigt–Reuss–Hill average, in log–log scale. For each material, the symbol is colored according to the Poisson's ratio *ν*: red indicates positive Poisson's ratio (*ν* > 0), and blue negative Poisson's ratio (*ν* < 0). The black line indicates the linear fit of data in log–log space: log_10_(*K*_VRH_) = 0.86 log_10_(*G*_VRH_) + 0.56, with variance score *R*^2^ = 0.63. The exponent of this power law (0.86) is relatively close to 1, the value expected if the relationship is linear. The green line corresponds to *K* = 2.2 *G*.

**Fig. 3:**
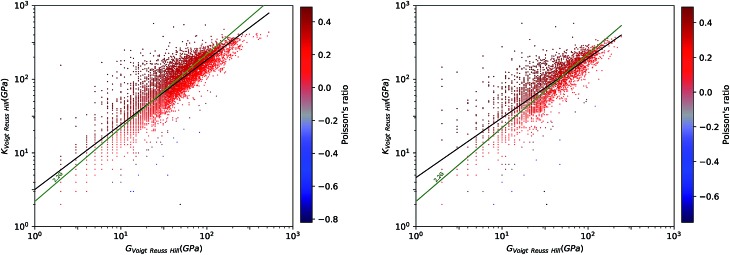
Plot of bulk modulus *K*_VRH_ against shear modulus *G*_VRH_, in log–log scale. Left: for 8050 experimentally synthesized inorganic compounds. Right: for 3714 hypothetical inorganic structures. Poisson's ratio is indicated by color scale. The black lines indicate a linear fit of data: log_10_(*K*_VRH_) = 0.88 log_10_(*G*_VRH_) + 0.50 (*R*^2^ = 0.97, left) and log_10_(*K*_VRH_) = 0.81 log_10_(*G*_VRH_) + 0.67 (*R*^2^ = 0.55, right). The green lines correspond to *K* = 2.2*G*.

To have a better view for the different trends of mechanical properties of inorganic compounds, we have plotted different elastic properties against each other, in logarithmic scale. For example, the ratio of the bulk to shear modulus has long been used to understand trends in the ductility of materials.^20^Fig. 2 provides a graphical representation of the bulk modulus *K vs.* shear modulus *G* for 11 764 inorganic structures (in the Voigt–Reuss-Hill average). As expected in classical models of mechanics, we find a broad correlation between the two quantities—the linear regression in log–log space has a variance score *R*^2^ = 0.63. These findings follow the same trend identified in the work of Jong *et al.* on a smaller database of 1181 inorganic structures.^19^ If we focus separately to the subsets of synthesized (8050) and hypothetical (3714) structures, presented in Fig. 3, we identify a clear difference: while the correlation is the same on average, the relationship is much more strictly obeyed in experimentally known materials (*R*^2^ = 0.97) than in hypothetical structures (*R*^2^ = 0.55). The same trends are also observed with different averaging schemes, Reuss and Voigt, as presented in ESI,† and appear to be a generic property of inorganic compounds. The difference observed between known and hypothetical structures is very significant, as it indicates that the “parameter space” of hypothetical structures is much wider than that of experimentally feasible materials. Thus, many suggested structures based on topological and energetic considerations could be mechanically unfeasible, a trend that was previously identified for zeolites^11^ but appears generalizable to all inorganic compounds.

**Fig. 4:**
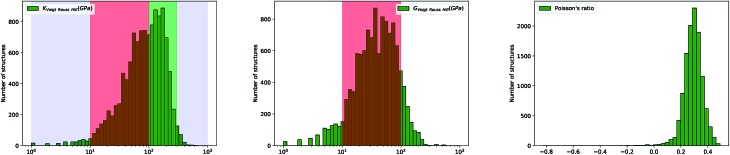
Histogram of Voigt–Reuss–Hill bulk modulus (in log scale), shear modulus (in log scale) and Poisson's ratio (in linear scale) for 11 764 inorganic compounds.

Furthermore, we have plotted on Fig. 2 the values of the average Poisson's ratio *ν* for each material, characterizing the amount of transversal expansion for a given uniaxial compression. It can be seen that overwhelming majority of inorganic materials display positive Poisson's ratio, as indicated by red points—those materials are also called *meiotic*. Overall, *ν* vary between –1 and 0.5, as can also be seen on the histogram in Fig. 4. The vast majority of structures have a value around *ν* ≈ 0.3, similar to most common materials such as steels and rigid polymers. Interestingly, we underline from Fig. 2 that *ν* tends to become negative with very small values of bulk modulus, relatively irrespective of the value of shear modulus—while for the highest *K* values (more than 100 GPa), all materials present in the Materials Project database display strongly positive Poisson's ratio. The overall trend observed is the same for the synthesized and to the hypothetical inorganic structures cases (see Fig. 3).

We find that, from the 11 764 compounds studied, only 75 materials exhibit negative average Poisson's ratio, *i.e.* only 1% of the structures. Auxeticity is thus a rare thing in inorganic solids in general, as has been established for zeolites, for examples.^25^ Those split relatively evenly between 45 synthesized and 30 hypothetical inorganic compounds. Their structures are presented in the ESI,† along with their material ID and a summary of their elastic properties. Such *ν* < 0 compounds, called *auxetic materials*, often display other anomalous elastic behaviour, and are of interest for their mechanical properties. This includes improved shear stiffness and shock absorption, with applications in the areas of body armour, increased-sensitivity piezoelectric composites, and fibre composites with greater pull-out resistance.^40^ This increased mechanical performance under constraint comes from the fact that these materials strongly densify under axial compression, leading in turn to increased resistance.

It is interesting to note that the mechanical properties of homogeneous, isotropic and linear materials can be uniquely determined by any two elastic moduli. This means, in particular, that *K*, *G* and *ν* are linked:^41^2
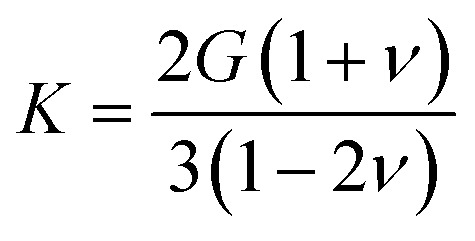



While anisotropic materials such as crystalline solids do not obey this law, we can however see that some relationship between *K*, *G* and *ν* appears to be hold in a statistical way. If we take *ν* = 0.3, the linear law would then be *K* = 2.2*G*, plotted in Fig. 2 and 3. This line actually lies close to the data points corresponding to materials with average Poisson's ratio close to 0.3. This makes an interesting link between the statistical behavior of a large number of anisotropic crystals, and the known behavior of isotropic materials—a conclusion similar to that reached by de Jong *et al.* on a smaller dataset.^19^

**Fig. 5:**
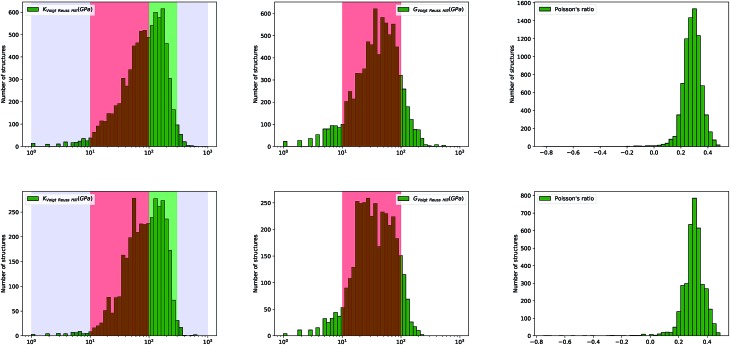
Histograms of log(*K*_VRH_), log(*G*_VRH_), and Poisson's ratio *ν* for 8050 synthesized (top) and 3714 hypothetical (bottom) inorganic compounds.

Looking further into the distribution of elastic properties, we plot in Fig. 4 the distribution of average elastic moduli, and split it between experimentally synthesized and hypothetical structures in Fig. 5—corresponding distributions for the Reuss and Voigt averaging schemes can be found in the ESI.† We see that *K* and *G* do not exceed 600 GPa for the selected inorganic compounds, and that shear modulus values fall mostly in the 10 to 100 GPa range (red-shaded area), which accounts for 90% of materials. *K* values are higher, with 60% of crystals studied in the 10–100 GPa range, and 35% ranging between 100 and 300 GPa (green-shaded area). Very soft (*K* < 10 GPa) and very stiff (*K* > 300 GPa) inorganic compounds are rare.

We note, finally, a marked difference in the distributions of elastic moduli when comparing synthesized and hypothetical inorganic structures. The distributions of *G* and *K* for hypothetical materials are wider, and almost bimodal—they feature more materials at low-to-intermediate modulus, near *G* ∼ 20 GPa and *K* ∼ 40 GPa (see Fig. 5). This is a confirmation that the space of mechanical properties spawned for hypothetical frameworks, enumerated by computational methods based on considerations of topology and formation enthalpy, is wider than the space of experimental structures. This indicates that our current methods for generating hypothetical structures are lacking, because they are not taken into consideration the mechanical properties in the evaluation of “feasibility” of hypothetical structures.‡
‡We note here that, of course, “experimentally feasible” structures are understood with respect to the current state of the published literature, and that structures that we classify as “unfeasible” today might be realized in the future, given the right synthetic conditions. Understanding this also opens the way to better evaluate such properties in the future.

### Anisotropy of the elastic properties

We now turn our attention to the analysis of anisotropy in the mechanical behavior of these materials. Indeed, as crystals are not isotropic solids, their elastic properties are tensorial in nature, and the resulting elastic moduli vary in space with the direction of the applied stress. This is of particular importance in screening of hypothetical materials for applications, as mechanical anisotropy has consequences on the macroscopic behavior and stability of materials.^42^,^43^ There is also a great interest in finding materials with atypical or “anomalous” mechanical behaviour, called mechanical metamaterials.^22^,^44^,^45^

We have thus analyzed with ELATE the directional dependence of elastic properties such Young's modulus (*E*), linear compressibility (*β*), shear modulus *G*, and Poisson's ratio *ν* of the 11 764 mechanically stable systems, of which 8050 have been experimentally synthesized and 3714 are hypothetical structures.^35^

**Fig. 6:**
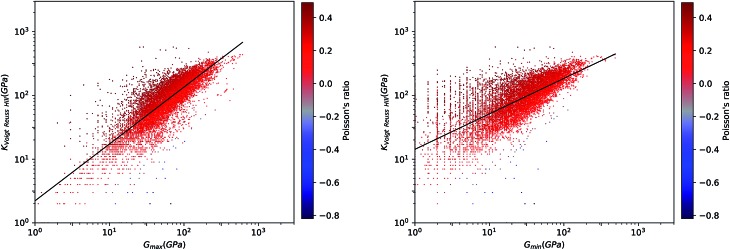
Voigt–Reuss–Hill bulk modulus *versus* maximum (left panel) and minimum (right panel) shear modulus for 11 764 inorganic compounds, in log–log scale. Poisson's ratio is indicated by color scale. Black lines indicate the linear fit of data: log_10_(*K*_VRH_) = 0.89 log_10_(*G*_max_) + 0.35 (*R*^2^ = 0.64, left) and log_10_(*K*_VRH_) = 0.56 log_10_(*G*_min_) + 1.15 (*R*^2^ = 0.64, right).

**Fig. 7:**
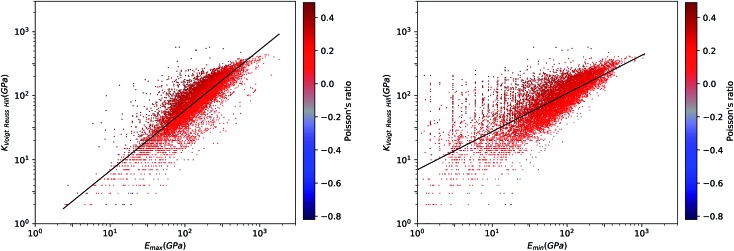
Voigt–Reuss–Hill bulk modulus *versus* maximum (left panel) and minimum (right panel) Young's modulus for 11 764 inorganic compounds, in log–log scale. Poisson's ratio is indicated by color scale. The black line indicates the linear fit of data: log_10_(*K*_VRH_) = 0.95 log_10_(*E*_max_) + 0.12 (*R*^2^ = 0.64, left) and log_10_(*K*_VRH_) = 0.60 log_10_(*E*_min_) + 0.84 (*R*^2^ = 0.64, right).

We first look at whether the remarkable correlation between *K* and *G* in the isotropic approximation still holds when considering extremal values, *i.e.* the maximal and minimal values of the directional shear modulus *G*. We plot in Fig. 6 the ratio (in log–log scale) of the VRH bulk modulus against shear moduli shear modulus. We find that *G*_min_ ranges from 0.03 GPa and 488 GPa, and *G*_max_ from 1 GPa to 611 GPa. We see in Fig. 6 that the isotropic bulk modulus, *K*_VRH_, displays a reasonably good correlation with both *G*_min_ and *G*_max_ for inorganic compounds. The same broad correlation is found between the isotropic behavior and the maximal and the minimal Young's modulus *E*, see Fig. 7—the Young's modulus quantifies the longitudinal strain resulting from a longitudinal stress in the same direction, see Fig. 1. So while individual crystals present anisotropy in their elastic properties, we conclude that there is *on average* a general trend underlying their behavior.

**Fig. 8:**
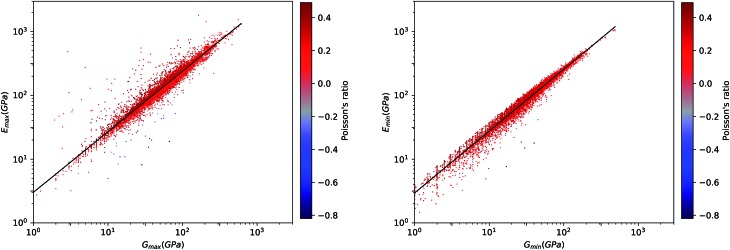
Plot of extremal values of Young's modulus *versus* shear modulus for 11 764 inorganic compounds, in log–log scale. Left: maximal values; right: minimal values. Poisson's ratio is indicated by color scale. The black lines indicate the linear fit of data: log_10_(*E*_max_) = 0.95 log_10_(*G*_max_) + 0.48 (*R*^2^ = 0.61, left) and log_10_(*E*_min_) = 0.97 log_10_(*G*_min_) + 0.47 (*R*^2^ = 0.96, right).

This same behavior can also be seen by looking directly at the correlation between *E* and *G*. In Fig. 8, we find a remarkable correlation between the maximal values of shear *G* and Young's *E* moduli (*R*^2^ ≈ 0.61). An even stronger correlation is found between the respective minimal values (*R*^2^ ≈ 0.96). This impressive correlation of *E*_min_ and *G*_min_ means that, although the inorganic compounds can have large anisotropy in their elastic properties, there exist a strong coupling between their softest modes of deformation in response to longitudinal and shear stress, respectively. This reinforces the conclusion reached in a previous, much more limited study of pure-silica zeolites (SiO_2_ polymorphs),^11^ by showing it holds for all inorganic structures, regardless of their chemical nature, geometry/topology, or porosity.

In order to better quantify the anisotropy of the mechanical properties of the materials in a systematic way, we defined their elastic anisotropy (*η*) defined as:3
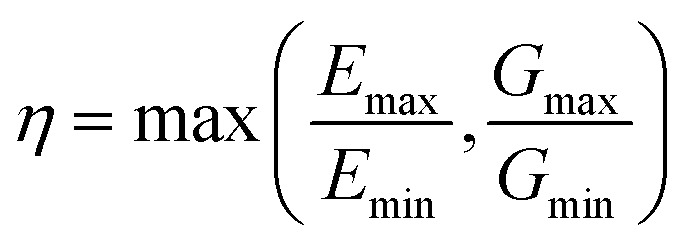



**Fig. 9:**
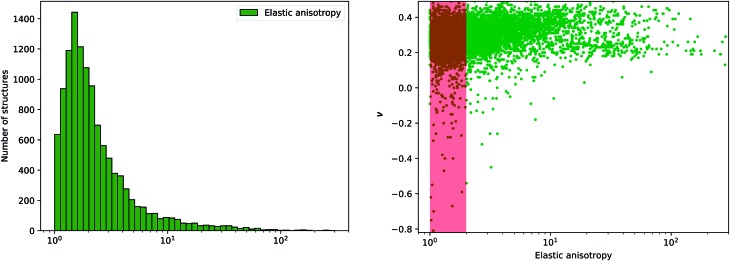
Left: Histogram of elastic anisotropy *η* (in log scale) for 11 764 inorganic compounds. Right: Poisson's ratio *ν versus* log(*η*), where the red area corresponds to anisotropy between 1 and 2.

This anisotropy is an important factor, as very high anisotropy is often correlated with a limited mechanical stability of the material—because of important internal stress upon deformation.^41^ Moreover, the elastic anisotropy has been proposed as a factor to determine the experimental feasibility of hypothetical structures.^11^ We plot in Fig. 9 the distribution of elastic anisotropy in inorganic materials. Interestingly, it clearly appears that considerable number of inorganic compounds, around 6000 materials which account for more than 50% of the structures, exhibit very low elastic anisotropy (*η* ≤ 2). The second half of considered materials have a higher elastic anisotropy, which can go up to 100 and above, making them mechanically fragile.

Furthermore, we have tried to identify a correlation between mechanical stability determined through elastic anisotropy and the elastic properties, and proposed in particular to look at the relationship between *η* and the average Poisson's ratio *ν*. We observe on Fig. 9 that most of materials with negative Poisson's ratio are located in the range of *η* ≤ 2. Thus, it is apparent that auxeticity is not associated with high anisotropy of the crystal, contrary to some previous hypotheses.^46^,^47^ We also conclude that although auxetic materials are scarce, they can be expected to be mechanically stable.

### Looking for anomalous properties

#### Auxetic materials

Auxetic materials, characterized by a negative Poisson ratio (*ν* < 0) in one or more directions of space, are a rare class of materials.^48^ They have attracted a widespread interest due to their exciting potential applications in extremely precise sensors, tougher ceramics and high-performance armor.^40^,^49^,^50^ As discussed above, analysis of the Materials Project elastic data in the isotropic approximation reveals only a limited number of inorganic materials with auxeticity (121 structures). When we analyzed these 126 structures further, we found out that only 75 are mechanically stable, highlighting the need for a full tensorial analysis of elastic properties. Previous work by Dagdelen *et al.* tried to extend this screening to the entire MP database (133 691 structures) by employing efficient screening algorithms based on known structural motifs to find auxetic materials from compounds available in MP database.^51^ The authors successively predicted 29 new materials with negative Poisson's ratio, and grouped structures with |*ν*| ≤ 0.1 (near-zero average Poisson's ratio) separately from auxetic materials, naming them “anepirretic”.

However, Poisson's ratio is a direction property, and there is interest in materials that feature anisotropic auxeticity.^25^ We have thus determined the maximal and the minimal values of *ν* for our set of 11 764 mechanical stable structures. Based on this, we could classify (following Siddorn *et al.*^25^) auxetic materials into two subclasses: the first one contains partially auxetic material (at least one direction with negative Poisson's ratio), and the second one is made of completely auxetic material (*ν* < 0 in all directions of space).

Of 11 764 inorganic materials, our search identified 30% of structures with auxetic behavior (3537 materials, whose full list of Material ID is available in ESI†). This is a significant number, showing that auxeticity *in some directions of space* is not actually a rare phenomenon. However, only 30 materials were found to be completely auxetic—these materials with *ν* < 0 in all directions being a subset of materials with < 0 in all directions being a subset of materials with 〈*ν*〉 < 0. In < 0. In Table 1, we list the properties of these 30 completely auxetic materials, of which 14 are hypothetical structures and 16 have been experimentally synthesized. We find that *ν*_max_ and *ν*_min_ range from –0.02 (Zr_2_TlC) to 0 (K_2_Rh_2_O_5_). In the same way, their average ). In the same way, their average 〈*ν*〉 varies from −0.05 (Li varies from –0.05 (Li_2_GaSb) to –0.82 (Cs_2_NaYF_6_). We also note that, for the 3507 partially auxetic materials, 44 structures present negative ). We also note that, for the 3507 partially auxetic materials, 44 structures present negative 〈*ν*〉 (and 3436 a positive average). We have visualized the structures of the 30 completely auxetic materials, but have not been able to identify common structural motifs that could be linked to their auxeticity in a general mechanism (like the JST zeolitic framework (and 3436 a positive average). We have visualized the structures of the 30 completely auxetic materials, but have not been able to identify common structural motifs that could be linked to their auxeticity in a general mechanism (like the JST zeolitic framework^25^). We publish in ESI† the structures, and suggest that classification according to topological or geometrical descriptors might identify common features in future work.

**Table 1:** List of completely auxetic materials in the Materials Project database, with extremal values of directional Poisson's ratio, and isotropic average

Material ID	Structure	Synthesized	*ν*_min_	*ν*_max_	〈*ν*〉
mp-1021516	K_2_Sn	No	–0.26	–0.20	–0.21
mp-9580	TlGaSe_2_	Yes	–0.94	–0.24	–0.59
mp-982773	Na_3_Tl	No	–0.50	–0.20	–0.4
mp-862769	RbGe_3_	No	–1.25	–0.17	–0.18
mp-974789	Rb_3_Sn	No	–0.75	–0.73	–0.62
mp-7621	KTcO_4_	Yes	–0.41	–0.04	–0.2
mp-36508	SnHgF_6_	No	–1.08	–0.10	–0.45
mp-15639	HgRhF_6_	Yes	–0.53	–0.14	–0.4
mp-999274	RbNaH_2_	Yes	–0.77	–0.47	–0.67
mp-697133	Cs_2_CaH_4_	Yes	–0.56	–0.32	–0.47
mp-27718	CsHgBr_3_	Yes	–0.15	–0.06	–0.12
mp-865080	NaCeAu_2_	No	–0.35	–0.29	–0.3
mp-13925	Cs_2_NaYF_6_	Yes	–0.85	–0.77	–0.82
mp-7961	Sr_3_SnO	Yes	–0.08	–0.08	–0.09
mp-989580	Cs_2_KNF_6_	No	–0.18	–0.07	–0.14
mp-989523	Rb_2_NaAsF_6_	No	–0.31	–0.20	–0.26
mp-4051	AlPO_4_	Yes	–0.58	–0.05	–0.28
mp-631316	Li_2_GaSb	No	–0.05	–0.05	–0.05
mp-866229	Ca_2_SnHg	No	–0.74	–0.65	–0.7
mp-2739	TeO_2_	Yes	–0.77	–0.37	–0.54
mp-989536	Cs_2_LiNF_6_	No	–0.78	–0.75	–0.75
mp-867920	K_2_Rh_2_O_5_	No	–0.57	–0.00	–0.27
mp-21200	PuGa_2_	Yes	–0.45	–0.07	–0.28
mp-989590	Ca_6_Sn_2_NF	No	–0.58	–0.53	–0.55
mp-20457	InP	Yes	–0.86	–0.77	–0.81
mp-1025524	Zr_2_TlC	Yes	–0.20	–0.02	–0.07
mp-1017566	GePbO_3_	Yes	–0.50	–0.26	–0.38
mp-1008282	Cr_3_Fe	Yes	–0.25	–0.04	–0.13
mp-978493	SiSnO_3_	No	–0.43	–0.04	–0.23
mp-10056	UCo_3_B_2_	Yes	–0.19	–0.06	–0.13

#### Negative linear compressibility

While volumetric compressibility is the inverse of bulk modulus, the linear compressibility *β* characterizes the response of a crystal along each direction of space, when compressed isotropically, *e.g.*, under hydrostatic pressure (see Fig. 1). While the volumetric compressibility must necessarily be positive (a criterion for thermodynamical stability), linear compressibility can be negative in one or more directions of space—meaning that upon isotropic compression, some linear dimensions expand. This behaviour is termed negative linear compressibility (NLC), and is considered an anomalous (and rare) mechanical property.^26^ NLC has been quite reported in some zeolites, MOFs structures, and other framework materials.^11^,^52^–^54^ NLC materials have a broad range of potential applications in designing pressure sensors and artificial muscles.^55^,^56^

We have analyze the Materials Project database to determine the minimal and the maximal values of linear compressibility for every materials. We find that *β*_min_ values range from –1890 TPa^–1^ to 2000 TPa^–1^ range, and the *β*_max_ values from 0.5 TPa^–1^ to 5073 TPa^–1^. We find that 357 inorganic compounds exhibit negative linear compressibility (meaning *β*_min_ < 0), accounting for 3% of the mechanically stable structures. The full list of the 357 NLC materials ID is available in the ESI.† Moreover, more than 50% of NLC materials are experimentally synthesized (238 structures), and most of them have not been mechanically characterized—and their NLC behaviour has not been discussed in previous literature.

Finally, we conclude that although NLC is indeed a rare phenomenon, there are more NLC structures than completely auxetic ones. This present large-scale screening of an inorganic materials database gives hope in the search for structures with anomalous mechanical properties, and will help scientists to identify materials with specific targeted properties—as well as inform a clear strategy to accelerate the discovery and design of novel structures with anomalous elastic behaviour.

## Conclusions and perspectives

In this work, we performed a large-scale exploration of the mechanical properties of 13 621 inorganic crystals, studying both their average mechanical properties (bulk and shear moduli) as well as probing the anisotropy of their elastic behavior. We have shown that general mechanical trends, which hold for isotropic (noncrystalline) materials at the macroscopic scale, also apply “on average” for inorganic crystals. By going beyond the isotropic approximation, we have highlighted the significance of the anisotropy of elastic properties, which can only be studied by a tensorial analysis of the full second-order elastic stiffness matrix. In particular, we point out a fundamental difference in the anisotropic elasticity between experimentally known materials and hypothetical frameworks, demonstrating that mechanics play a role in the experimental feasibility of inorganic compounds.

In addition to looking at those general trends, we have further identified crystalline materials with anomalous elastic properties, such as negative Poisson's ratio and negative linear compressibility. We have quantified, for the first time in a study of that scale, exactly how frequently those properties are encountered: negative linear compressibility is found in 3% of inorganic materials, while partial auxeticity is found in 30%. Total auxeticity is the rarest of these phenomena, being observed in 0.3% of crystals studied…although those 30 totally auxetic materials represent a huge leap up from unique example identified to date (the JST zeolite framework).^25^ This better understanding of the rarity of various anomalous properties in crystals is a necessary first step to enable the design of new mechanical metamaterials, based not on a designed micro- or macro-structure, but on a nanoscopic crystalline framework.^27^ Experimental and computational characterization of framework materials has, so far, taken place in a very step-by-step fashion, where a few materials are characterized in depth in each study.^18^,^57^,^58^ Based on the knowledge gained in this study, we now look to apply high-throughput screening methods to larger databases of materials,^59^ in order to accelerate discovery of mechanical metamaterials based not on their complete elastic characterization, but on descriptors such as their structural motifs, composition, topology, *etc.*

## Conflicts of interest

There are no conflicts to declare.

## Supplementary Material

Supplementary information
