# Supplementary material for: Systematic exploration of the mechanical properties of 13 621 inorganic compounds
Source: Chem Sci. 2019 Jul 31;10(37):8589–99. doi: 10.1039/c9sc01682a (PMC6844276; doi:10.1039/c9sc01682a)
Supplement: Supplementary file 1 [file SC-010-C9SC01682A-s001.pdf]

Electronic Supplementary Material (ESI) for Chemical Science.
This journal is © The Royal Society of Chemistry 2019

Supporting Information for: Systematic Exploration of the Mechanical Properties of 13,621 Inorganic Compounds

Siwar Chibani* and François-Xavier Coudert*

Chimie ParisTech, PSL University, CNRS, Institut de Recherche de Chimie Paris, 75005 Paris, France

E-mail: siwar.chebbi@chimieparistech.psl.eu; fx.coudert@chimieparistech.psl.eu

Elastic properties in the isotropic approximation

Table 1: List of the 13 materials presenting K and G values more then 10^3 GPa.

material ID	$\langle\mu\rangle$	G_{Reuss}	G_{Voigt}	G_{VRH}	K_{Reuss}	K_{Voigt}	K_{VRH}
mp-13181	-0.76	17.0	1984.0	1000.0	63.0	63.0	63.0
mp-973434	-0.27	1026.0	21.0	524.0	167.0	167.0	167.0
mp-7723	-0.6	1114.0	24.0	569.0	67.0	69.0	68.0
mp-1008688	-0.66	1238.0	8.0	623.0	60.0	60.0	60.0
mp-862714	-0.32	1257.0	110.0	684.0	190.0	191.0	190.0
mp-976406	-0.41	1708.0	30.0	869.0	188.0	188.0	188.0
mp-999135	-0.52	1926.0	44.0	985.0	141.0	170.0	155.0
mp-754542	-0.76	1995.0	11.0	1003.0	63.0	64.0	64.0
mp-702	-0.96	2457.0	7.0	1232.0	12.0	12.0	12.0
mp-73	-0.7	2562.0	13.0	1287.0	105.0	105.0	105.0
mp-999002	-0.52	2871.0	21.0	1446.0	224.0	224.0	224.0
mp-864844	-0.83	6593.0	25.0	3309.0	144.0	144.0	144.0
mp-631409	-0.79	10574.0	31.0	5303.0	289.0	289.0	289.0

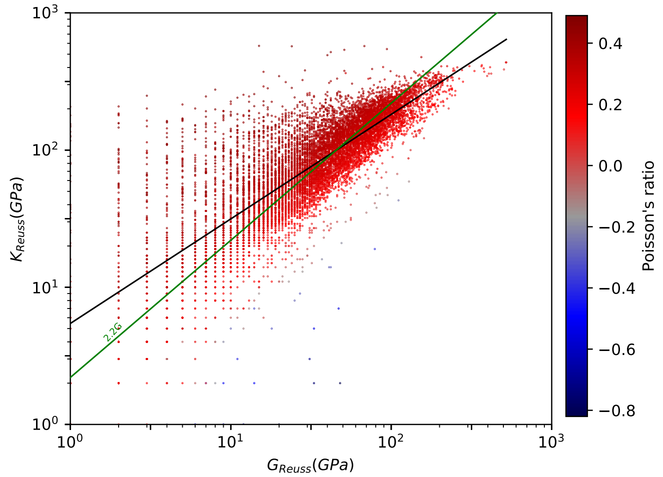

Figure 1: Plot of bulk modulus K_{R} against shear modulus G_{R} for 11,764 inorganic compounds from the Material Project database, in the Reuss average, in log–log scale. For each material, the symbol is filled according to the Poisson's ratio ν: red indicates positive Poisson's ratio ($\nu > 0$), and blue negative Poisson's ratio ($\nu < 0$). The black line indicates the linear fit of data (log_{10} (K_R)= 0.76 * log_{10} (G_R)+0.74) with an variance score R^2 of 0.67. The green line corresponds to $K = 2.2G$.

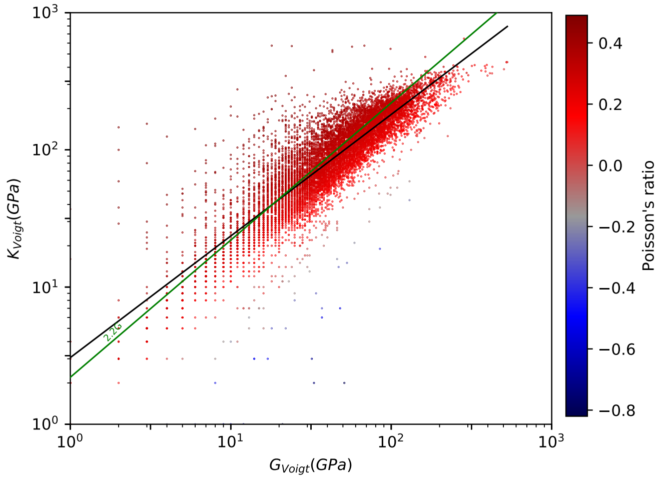

Figure 2: Plot of bulk modulus K_{V} against shear modulus G_{V} for 11,764 inorganic compounds from the Material Project database, in the Voigt average, in log–log scale. For each material, the symbol is filled according to the Poisson's ratio ν: red indicates positive Poisson's ratio ($\nu > 0$), and blue negative Poisson's ratio ($\nu < 0$). The black line indicates the linear fit of data (log_{10} (K_V)= 0.89 * log_{10} (G_V)+0.49) with an variance score R^2 of 0.60. The green line corresponds to $K = 2.2G$.

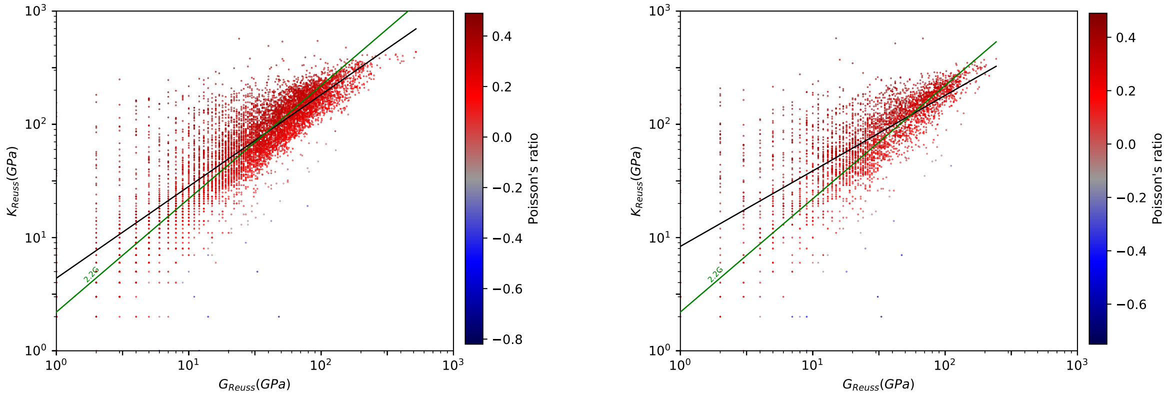

Figure 3: Plot of bulk modulus K_{R} against shear modulus G_{R}, in log–log scale. Left: for 8,050 experimentally synthesized inorganic compounds. Right: for 3,714 hypothetical inorganic structures. Poisson's ratio is indicated by color scale. Bar plots color is related to the Poisson ratio value: red points correspond to the positive Poisson ratio while the white and the blue points correspond to the negative one. Bulk modulus, shear modulus and Poisson ratio values are extracted from Material Project database. The black lines indicate a linear fit of data (log_{10} (K)= 0.81 * log_{10} (G_R)+0.64) with an variance score R^2 of 0.88 and log_{10} (K_R)= 0.67 * log_{10} (G_R)+0.92 with an variance score R^2 of 0.55 for experimentally and hypothetical inorganic structures, respectively). The green lines correspond to $K = 2.2G$.

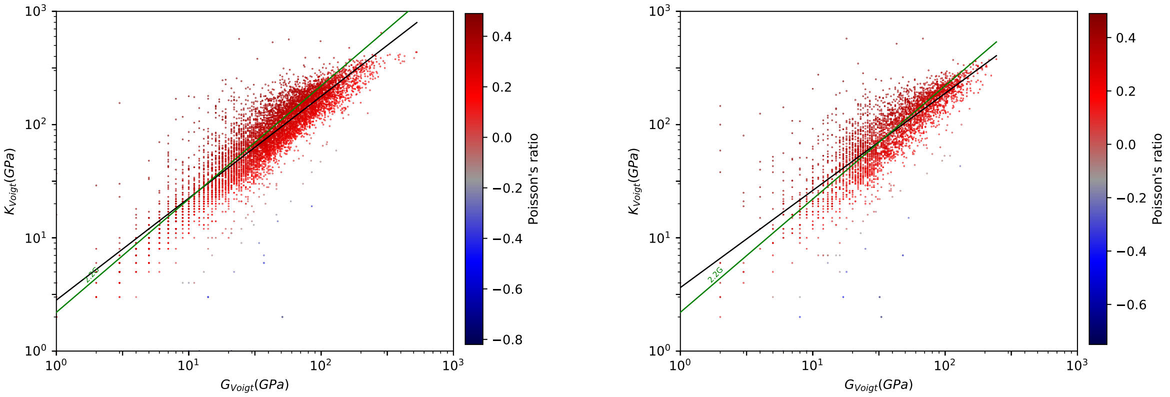

Figure 4: Plot of bulk modulus K_{V} against shear modulus G_{V}, in log–log scale. Left: for 8,050 experimentally synthesized inorganic compounds. Right: for 3,714 hypothetical inorganic structures. Poisson's ratio is indicated by color scale. Bar plots color is related to the Poisson ratio value: red points correspond to the positive Poisson ratio while the white and the blue points correspond to the negative one. Bulk modulus, shear modulus and Poisson ratio values are extracted from Material Project database. The black lines indicate a linear fit of data (log_{10} (K_V)= 0.90 * log_{10} (G_V)+0.45) with an variance score R^2 of 0.97 and log_{10} (K_V)= 0.87 * log_{10} (G_V)+0.53 with an variance score R^2 of 0.52 for experimentally and hypothetical inorganic structures, respectively). The green lines correspond to $K = 2.2G$.

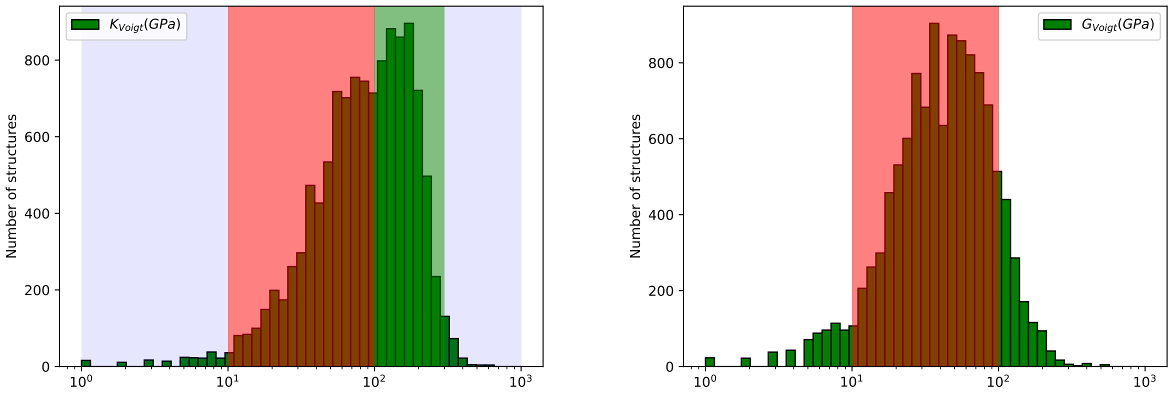

Figure 5: Histogram of log Voigt bulk modulus and log Voigt shear modulus for 11,764 inorganic compounds. The corresponding values are extracted from the Material Project database. The different areas colors is related to the distribution of materials relative to their respective values.

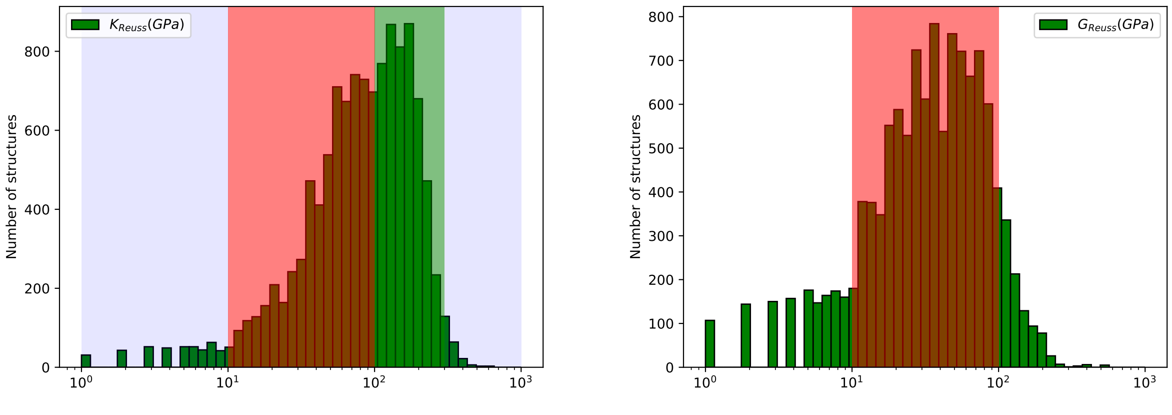

Figure 6: Histogram of log Reuss bulk modulus and log Reuss shear modulus for 11,764 inorganic compounds. The corresponding values are extracted from the Material Project database. The different areas colors is related to the distribution of materials relative to their respective values.

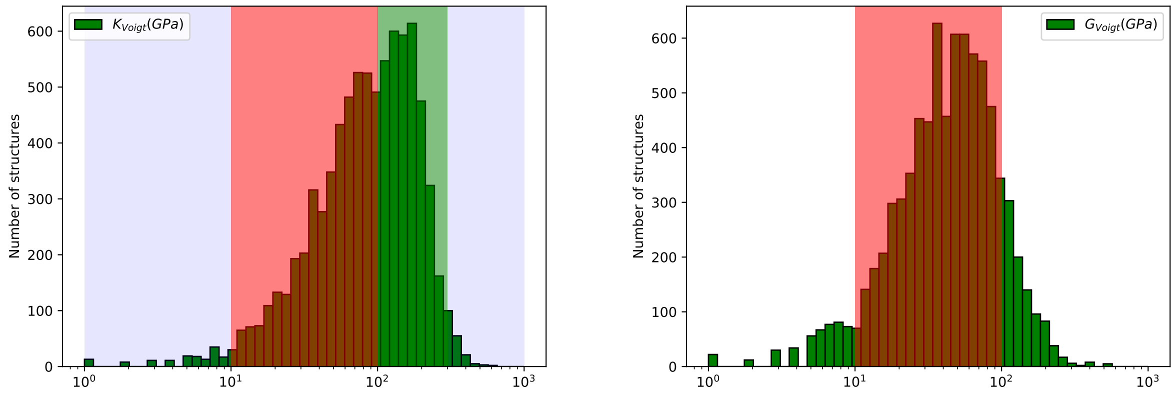

Figure 7: Histogram of log Voigt bulk modulus and log Voigt shear modulus for 8,050 synthesized inorganic compounds. The corresponding values are extracted from the Material Project database. The different areas colors is related to the distribution of materials relative to their respective values.

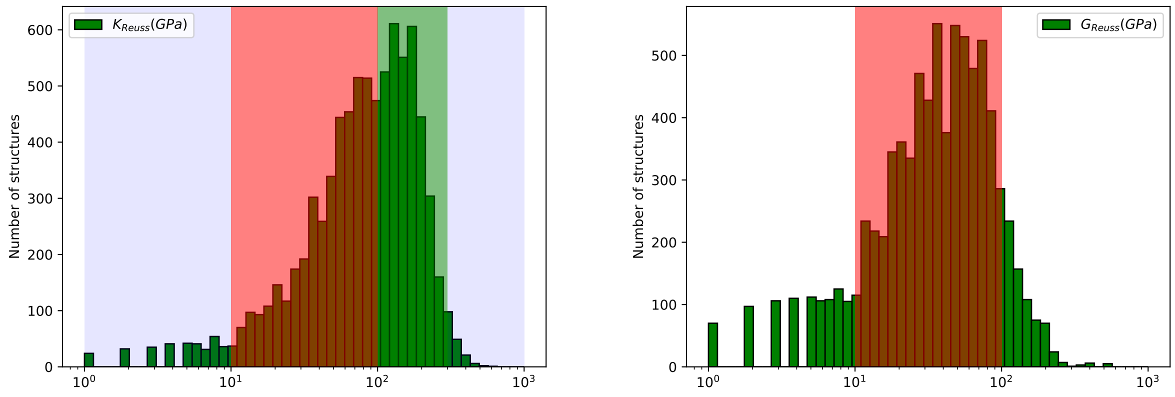

Figure 8: Histogram of log Reuss bulk modulus and log reuss shear modulus for 8,050 synthesized inorganic compounds. The corresponding values are extracted from the Material Project database. The different areas colors is related to the distribution of materials relative to their respective values.

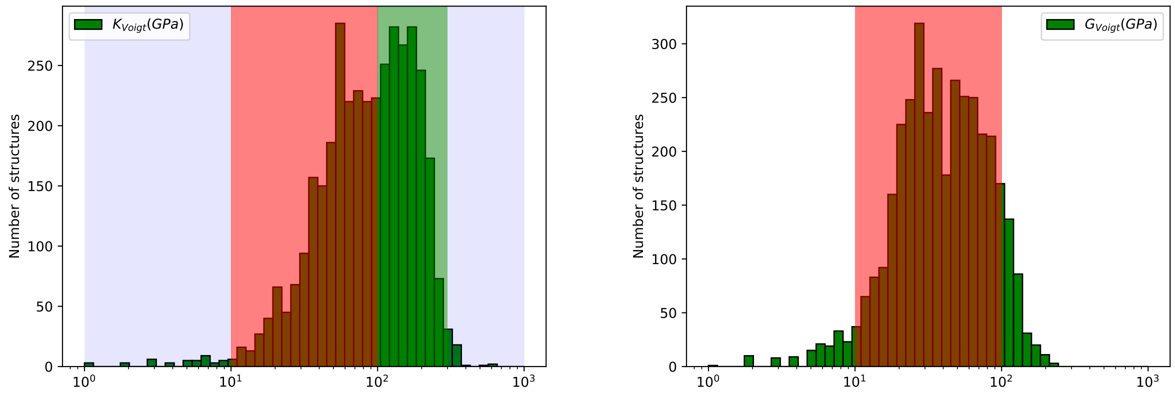

Figure 9: Histogram of log Voigt bulk modulus and log Voigt shear modulus for 3,714 hypothetical inorganic compounds. The corresponding values are extracted from the Material Project database. The different areas colors is related to the distribution of materials relative to their respective values.

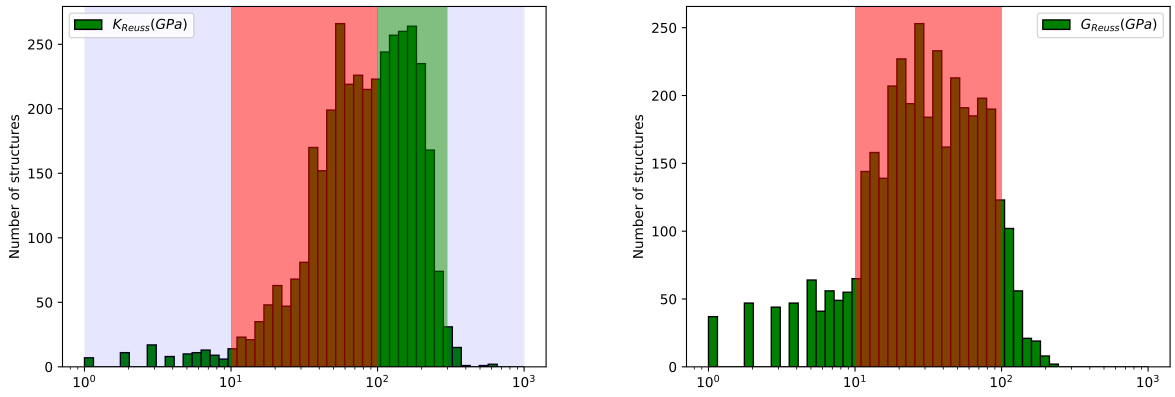

Figure 10: Histogram of log Reuss bulk modulus and log Reuss shear modulus for 3,714 hypothetical inorganic compounds. The corresponding values are extracted from the Material Project database. The different areas colors is related to the distribution of materials relative to their respective values.

Table 2: List of the 75 materials presenting negative Poisson's ratio with their relative elastic properties values in the isotropic approximation.

material ID	formula	$\langle\mu\rangle$	G_{Reuss}	G_{Voigt}	G_{VRH}	K_{Reuss}	K_{Voigt}	K_{VRH}
mp-7615	Rb_3TlF_6	-0.85	7.0	10.0	9.0	0.0	0.0	0.0
mp-13925	Cs_2NaYF_6	-0.82	20.0	21.0	21.0	1.0	1.0	1.0
mp-20457	InP	-0.81	48.0	51.0	49.0	2.0	2.0	2.0
mp-989536	Cs_2LiNF_6	-0.75	33.0	33.0	33.0	2.0	2.0	2.0
mp-866229	Ca_2SnHg	-0.7	31.0	32.0	31.0	3.0	3.0	3.0
mp-999274	$RbNaH_2$	-0.67	12.0	12.0	12.0	1.0	1.0	1.0
mp-974789	Rb_3Sn	-0.62	9.0	9.0	9.0	1.0	1.0	1.0
mp-9580	$TlGaSe_2$	-0.59	6.0	10.0	8.0	1.0	1.0	1.0
mp-989590	Ca_6Sn_2NF	-0.55	47.0	48.0	47.0	7.0	7.0	7.0
mp-2739	TeO_2	-0.54	33.0	37.0	35.0	5.0	6.0	5.0
mp-697133	Cs_2CaH_4	-0.47	14.0	14.0	14.0	2.0	3.0	3.0
mp-36508	$SnHgF_6$	-0.45	9.0	17.0	13.0	2.0	3.0	3.0
mp-982773	Na_3Tl	-0.4	7.0	8.0	8.0	2.0	2.0	2.0
mp-15639	$HgRhF_6$	-0.4	11.0	14.0	13.0	3.0	3.0	3.0
mp-1017566	$GePbO_3$	-0.38	79.0	85.0	82.0	19.0	19.0	19.0
mp-8224	$CaSnF_6$	-0.32	14.0	37.0	26.0	7.0	7.0	7.0
mp-865080	$NaCeAu_2$	-0.3	18.0	18.0	18.0	5.0	5.0	5.0
mp-21200	$PuGa_2$	-0.28	42.0	47.0	45.0	14.0	14.0	14.0
mp-867920	$K_2Rh_2O_5$	-0.27	41.0	53.0	47.0	14.0	15.0	15.0
mp-989523	Rb_2NaAsF_6	-0.26	25.0	25.0	25.0	8.0	8.0	8.0
mp-8539	SrSnP	-0.26	10.0	22.0	16.0	5.0	5.0	5.0
mp-975380	$RbCdO_3$	-0.26	1.0	6.0	3.0	1.0	1.0	1.0
mp-978493	$SiSnO_3$	-0.23	111.0	130.0	121.0	43.0	43.0	43.0
mp-1021516	K_2Sn	-0.21	4.0	4.0	4.0	1.0	1.0	1.0

Table 2: List of the 75 materials presenting negative Poisson's ratio with their relative elastic properties values in the isotropic approximation.

material ID	formula	$\langle\mu\rangle$	G_{Reuss}	G_{Voigt}	G_{VRH}	K_{Reuss}	K_{Voigt}	K_{VRH}
mp-7621	$KTcO_4$	-0.2	9.0	10.0	10.0	4.0	4.0	4.0
mp-6945	SiO_2	-0.2	37.0	46.0	41.0	16.0	16.0	16.0
mp-999052	Ti_2MnAl	-0.19	49.0	59.0	54.0	21.0	21.0	21.0
mp-862769	$RbGe_3$	-0.18	8.0	13.0	10.0	2.0	6.0	4.0
mp-27294	Ca_3AsBr_3	-0.15	28.0	31.0	30.0	13.0	13.0	13.0
mp-36248	H_4BrN	-0.15	8.0	9.0	8.0	4.0	4.0	4.0
mp-989580	Cs_2KNF_6	-0.14	23.0	23.0	23.0	11.0	11.0	11.0
mp-1008752	CeTe	-0.14	15.0	25.0	20.0	9.0	9.0	9.0
mp-975383	$RbCa_3$	-0.14	4.0	8.0	6.0	3.0	3.0	3.0
mp-1008282	Cr_3Fe	-0.13	81.0	85.0	83.0	38.0	38.0	38.0
mp-10056	UCo_3B_2	-0.13	129.0	130.0	129.0	59.0	59.0	59.0
mp-2639	Na_3N	-0.13	7.0	13.0	10.0	5.0	5.0	5.0
mp-27718	$CsHgBr_3$	-0.12	15.0	15.0	15.0	7.0	7.0	7.0
mp-774922	Ti_3TeO_8	-0.12	12.0	15.0	13.0	5.0	7.0	6.0
mp-989532	Rb_2TlInF_6	-0.11	13.0	16.0	14.0	7.0	7.0	7.0
mp-6074	KRb_2TiF_6	-0.11	52.0	73.0	63.0	30.0	30.0	30.0
mp-1017467	$CaMnO_3$	-0.11	95.0	99.0	97.0	47.0	47.0	47.0
mp-1006886	CeS	-0.1	28.0	30.0	29.0	14.0	14.0	14.0
mp-22817	$TaTe_4$	-0.1	21.0	25.0	23.0	12.0	12.0	12.0
mp-776651	Mn_3NiO_4	-0.09	70.0	73.0	71.0	35.0	37.0	36.0
mp-7961	Sr_3SnO	-0.09	21.0	21.0	21.0	11.0	11.0	11.0
mp-1019259	SrSnHg	-0.09	25.0	27.0	26.0	13.0	15.0	14.0
mp-962075	SrMgSn	-0.09	4.0	16.0	10.0	5.0	5.0 5	.0
mvc-5096	WO_3	-0.08	80.0	109.0	95.0	47.0	53.0	50.0

Table 2: List of the 75 materials presenting negative Poisson's ratio with their relative elastic properties values in the isotropic approximation.

material ID	formula	$\langle\mu\rangle$	G_{Reuss}	G_{Voigt}	G_{VRH}	K_{Reuss}	K_{Voigt}	K_{VRH}
mp-771798	WO_3	-0.08	85.0	95.0	90.0	48.0	48.0	48.0
mp-570223	$CsGeBr_3$	-0.08	20	21	20	11	11	11
mp-1025524	Zr_2TlC	-0.07	79.0	84.0	81.0	41.0	48.0	44.0
mp-775001	V_3FeO_8	-0.06	11.0	13.0	12.0	6.0	7.0	7.0
mp-8936	SnSe	-0.06	6.0	14.0	10.0	2.0	10.0	6.0
mp-975425	$RbAsO_3$	-0.06	94.0	99.0	96.0	53.0	53.0	53.0
mp-571124	Cs_2KScCl_6	-0.06	5.0	11.0	8.0	4.0	4.0	4.0
mp-1002083	CsKICl	-0.06	4.0	5.0	4.0	2.0	3.0	3.0
mp-554089	SiO_2	-0.06	33.0	38.0	35.0	20.0	20.0	20.0
mp-21139	Mn_2SnS_4	-0.06	32.0	37.0	35.0	15.0	24.0	19.0
mp-6058	K_2NaScF_6	-0.05	26.0	28.0	27.0	16.0	16.0	16.0
mp-985285	$CoHg_3$	-0.05	12.0	13.0	12.0	7.0	7.0	7.0
mp-764744	V_2OF_5	-0.05	28.0	46.0	37.0	16.0	26.0	21.0
mp-24292	Sr_2H_6Ru	-0.05	30.0	31.0	30.0	17.0	17.0	17.0
mp-631316	Li_2GaSb	-0.05	30.0	30.0	30.0	17.0	17.0	17.0
mp-972119	$SrCaTl_2$	-0.05	38.0	40.0	39.0	23.0	23.0	23.0
mp-766784	V_3CoO_8	-0.04	10.0	12.0	11.0	6.0	7.0	6.0
mp-865755	$YbCeHg_2$	-0.04	22.0	24.0	23.0	14.0	14.0	14.0
mp-561543	BeF_2	-0.04	14.0	14.0	14.0	8.0	8.0	8.0
mp-9056	$ZrPdF_6$	-0.03	10.0	21.0	15.0	7.0	12.0	9.0
mp-557397	K_2MnF_6	-0.03	15.0	16.0	16.0	10.0	10.0	10.0
mp-1007778	TlN	-0.03	109.0	110.0	110.0	67.0	67.0	67.0
mp-6930	SiO_2	-0.03	42.0	46.0	44.0	27.0	27.0	27.0
mp-6204	Cs_2NaScF_6	-0.02	30.0	30.0	30.0	19.0	19.0	19.0

Table 2: List of the 75 materials presenting negative Poisson's ratio with their relative elastic properties values in the isotropic approximation.

material ID	formula	$\langle \mu \rangle$	G_{Reuss}	G_{Voigt}	G_{VRH}	K_{Reuss}	K_{Voigt}	K_{VRH}
mp-28698	Na_4IrO_4	-0.02	16.0	28.0	22.0	6.0	21.0	14.0
mp-557837	SiO_2	-0.01	27.0	34.0	30.0	16.0	23.0	19.0
mp-11390	LiGaSi	-0.01	46.0	46.0	46.0	30.0	30.0	30.0

Anisotropy of the elastic properties

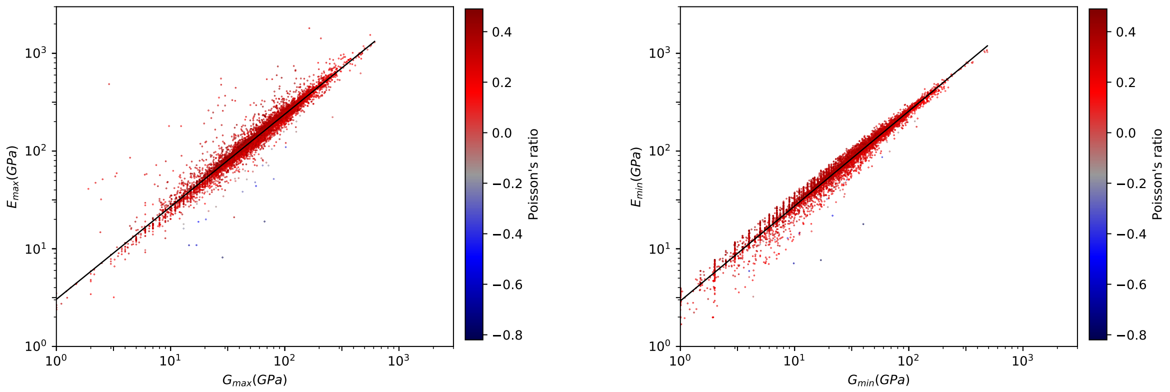

Figure 11: Left (Right) log maximum (minimum) young modulus versus log maximum (minimum) shear modulus for 8,050 synthesized inorganic compounds. Poisson's ratio is indicated by color scale. Bar plots color is related to the Poisson ratio value: red points correspond to the positive Poisson ratio while the white and the blue points correspond to the negative one. The black line indicates the linear fit of data (log_{10} (E_{max})= 0.95 * log_{10} (G_{max})+0.48) with an variance score R^2 of 0.81 and log_{10} (E_{min})= 0.97 * log_{10} (G_{min})+0.47 with an variance score R^2 of 0.58, respectively). Poisson's ratio, maximum and minimum shear and young modulus values are determined through ELATE application.

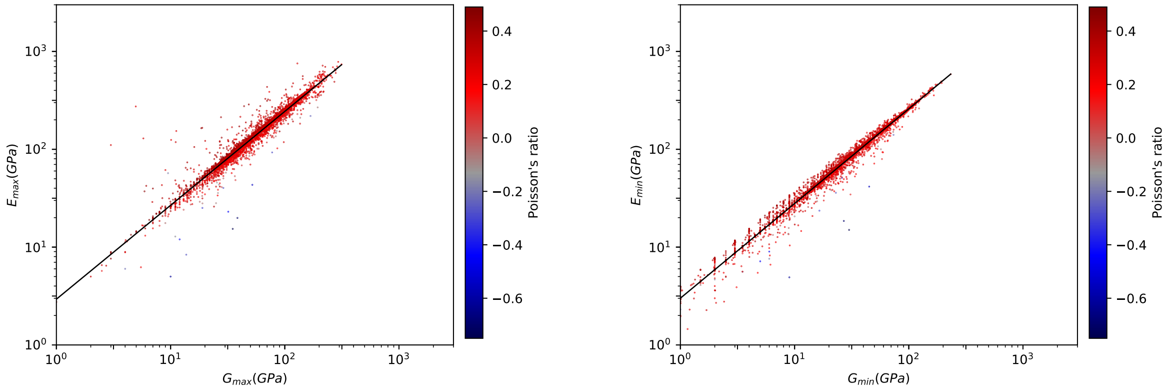

Figure 12: Left (Right) log maximum (minimum) young modulus versus log maximum (minimum) shear modulus for 3,714 hypothetical inorganic compounds. Poisson's ratio is indicated by color scale. Bar plots color is related to the Poisson ratio value: red points correspond to the positive Poisson ratio while the white and the blue points correspond to the negative one. The black line indicates the linear fit of data (log_{10} (E_{max})= 0.96 * log_{10} (G_{max})+0.47) with an variance score R^2 of 0.55 and log_{10} (E_{min})= 0.97 * log_{10} (G_{min})+0.48 with an variance score R^2 of 0.95, respectively). Poisson's ratio, maximum and minimum shear and young modulus values are determined through ELATE application.

Anomalous mechanical properties

The list of materials ID for the 357 materials presenting negative linear compressibility (NLC) and the 3,537 materials presenting negative minimum Poisson's ratio can be found in the attached spreadsheet.
